# Knockdown of NCOR2 Inhibits Cell Proliferation via BDNF/TrkB/ERK in NF1-Derived MPNSTs

**DOI:** 10.3390/cancers14235798

**Published:** 2022-11-24

**Authors:** Yuehua Li, Manhon Chung, Rehanguli Aimaier, Chengjiang Wei, Wei Wang, Lingling Ge, Beiyao Zhu, Zizhen Guo, Mingyang Wang, Yihui Gu, Haibing Zhang, Qingfeng Li, Zhichao Wang

**Affiliations:** 1 Department of Plastic and Reconstructive Surgery, Shanghai Ninth People’s Hospital, Shanghai Jiao Tong University School of Medicine, Shanghai 200023, China; 2 CAS Key Laboratory of Nutrition, Metabolism and Food Safety, Shanghai Institute of Nutrition and Health, University of Chinese Academy of Sciences, Chinese Academy of Sciences, Shanghai 100864, China

**Keywords:** neurofibromatosis type 1, MPNST, NCOR2, BDNF, MAPK, cell proliferation

## Abstract

**Simple Summary:**

Malignant peripheral nerve sheath tumours (MPNSTs) are highly aggressive and invasive peripheral soft-tissue sarcomas that typically develop in the context of neurofibromatosis type 1 (NF1). Compared with sporadic MPNSTs, patients with NF1-derived MPNSTs are younger and have a worse prognosis. The aim of our study is to further identify potential targets for the treatment of NF1-derived MPNSTs based on existing therapies. We found that the nuclear receptor corepressor 2 (NCOR2) could regulate ERK activation through the brain-derived neurotrophic factor (BDNF)/TrkB pathway, thereby affecting the growth of NF1-derived MPNSTs. This finding may provide new drug targets and combined drug therapy strategies for the clinical treatment of NF1-derived MPNSTs.

**Abstract:**

(1) Background: malignant peripheral nerve sheath tumours (MPNSTs) are aggressive Schwann cell-derived sarcomas with dismal prognoses. Previous studies have shown that nuclear receptor corepressor 2 (NCOR2) plays a vital role in neurodevelopment and in various tumours. However, the impact of NCOR2 on the progression of MPNST remains unclear. (2) Methods: by GEO database, MPNST tissue microarray, and NF1-related tumour tissues and cell lines were used to explore NCOR2 expression level in the MPNSTs. The role and mechanism of NCOR2 in NF1-derived MPNSTs were explored by experiments in vivo and in vitro and by transcriptome high-throughput sequencing. (3) Results: NCOR2 expression is significantly elevated in NF1-derived MPNSTs and is associated with patient 10-year survival time. Knockdown of NCOR2 suppressed NF1-derived MPNST cell proliferation by blocking the cell cycle in the G0/G1 phase. Moreover, decreased NCOR2 expression could down-regulate MAPK signal activity through the BDNF/TrkB pathway. (4) Conclusions: our findings demonstrated that NCOR2 expression is significantly elevated in NF1-derived MPNSTs. NCOR2 knockdown can inhibit NF1-derived MPNST cell proliferation by weakened BDNF/TrkB/ERK signalling. Targeting NF1-derived MPNSTs with TrkB inhibitors, or in combination with ERK inhibitors, may be a novel therapeutic strategy for clinical trials.

## 1. Introduction

Malignant peripheral nerve sheath tumours (MPNSTs) are highly aggressive and invasive peripheral nerve-associated soft-tissue sarcomas that typically develop in the context of neurofibromatosis type 1 (NF1) [1,2]. NF1 is an autosomal dominant cancer predisposition syndrome caused by mutations in the NF1 gene that afflicts approximately one in 3000 individuals worldwide [3]. Among NF1 patients, 20% to 50% develop benign plexiform neurofibromas (PNFs) [4]. It is not negligible that 8–13% of PNFs will further develop into MPNSTs, accounting for half of the total MPNST cases [5]. Moreover, NF1 patients develop MPNSTs at a significantly younger age, and the survival duration of NF1-derived MPNST patients is greatly reduced [6]. This dismal prognosis is largely caused by the tendency of NF1-derived MPNSTs to be larger and deeper due to rapid growth [7]. Therefore, elucidation of the mechanisms for NF1-derived MPNST growth is vital.

The genomic characterization of MPNST cohorts revealed some genes that are frequently dysregulated in MPNSTs, including the loss of function of NF1, SUZ12, or EED and the deletion of the CDKN2A/B locus [8]. As the necessary genetic alteration in NF1-derived MPNSTs, biallelic inactivation of NF1 leads to the aberrant amplification of Ras and downstream mitogen-activated protein kinase (MAPK) oncogenic signalling, suggesting that molecules in this pathway are powerful potential therapeutic targets [9]. At present, MEK inhibitor (MEKi) selumetinib has been approved for use in PNFs [10]. However, the efficacy of MEKi monotherapy was relatively limited in MPNSTs [11,12]. Therefore, further searching for new therapeutic targets for MPNSTs on the basis of existing surgical and drug therapy and rational design of combined drug delivery regimens has become one of the clinical and basic concerns.

Nuclear receptor corepressor 2 (NCOR2) belongs to the family of transcriptional corepressors and is widely expressed in human tissues. It is often recruited to nuclear and nonnuclear receptors in a large repressing complex containing histone deacetylases [13] that regulates the expression of different genes to maintain the development of multiple systems [14,15,16]. In addition, NCOR2 is closely related to the development of multiple tumours and has been considered a potential therapeutic target. Notably, the roles of NCOR2 as a pro-oncogenic or tumour suppressor depend on the tumour types. In breast cancer, Karmakar S et al. reported that NCOR2 can promote SRC3-dependent gene expression and elevate cyclin D1 levels, promoting cell proliferation [17]. Moreover, in several large studies evaluating human breast tumours, higher NCOR2 protein levels correlated with poor prognosis [18,19,20]. Elevated NCOR2 levels are common in prostate cancer cells, resulting in promoting cell proliferation [21,22]. However, Long MD et al. pointed out that high NCOR2 expression is favourable for androgen deprivation therapy in prostate cancer [23]. Mori T. et al. revealed that high NCOR2 levels in multiple myeloma patients facilitate multidrug therapy [24]. In conclusion, significant heterogeneity exists in the roles of NCOR2 in different types of tumours.

Furthermore, abnormal expression of NCOR2 in the neurodevelopmental system can also promote tumourigenesis. Campos B et al. found strong nuclear expression of NCOR2 in 283 astrocytic gliomas, which is related to tumour proliferation and differentiation [25]. However, the biological significance of NCOR2, as a pro- or anti-tumourigenic molecule in MPNSTs, especially in the NF1-derived MPNSTs, remains to be determined. 

## 2. Materials and Methods

### 2.1. Cell Culture

One normal human Schwann cell (HSC) line and three PNF cell lines (ipNF05.5, ipNF05.5 mixed clones, and ipNF9511.bc) were purchased from the American Type Culture Collection (ATCC, Rockville, MD, USA). Four NF1-derived MPNST cell lines (ST8814, T265, S462, and S462TY) and one sporadic MPNST cell line (STS26T) were kindly donated by Prof. Vincent Keng [26] and Prof. Jilong Yang [27]. The cells were cultured in Dulbecco’s modified Eagle’s medium (DMEM, Gibco, New York, NY, USA), supplemented with 10% foetal bovine serum (FBS, HyClone, UT, USA), 100 U/mL penicillin and 100 µg/mL streptomycin, maintained in a humidified atmosphere containing 5% CO_2_ at 37 °C, and confirmed to be negative for mycoplasma every 3 months.

### 2.2. Patients and Specimens

From 2015 to 2018, postoperative tissues of 49 MPNST patients were paraffin-embedded for the construction of a tissue microarray and immunohistochemistry, including NF1-derived (19) and sporadic (30) tumours. For NF1-derived nerve sheath tumours, different tumour types were diagnosed according to tumour cell atypia, neurofibroma structural loss, cell density, mitosis, and necrosis, as detailed in Table S1. Tumour differentiation, mitosis, and necrosis were scored according to the FNCLCC Histological Grading System in sporadic MPNSTs. A total score of 2–3 is classified as low-grade, and >3 is classified as high-grade (see Table S2 for details). Each patient’s clinical information was obtained from the hospital’s electronic medical system. Since 20 of the 49 patients were lost to follow-up, only 29 were included in the survival analysis, as previously described [28].

Fresh human PNF and NF1-derived MPNST tissues were obtained from surgical resection at Shanghai Ninth People’s Hospital (Shanghai, China). The study was approved by the Ethics Committee of Shanghai Ninth People’s Hospital, Shanghai Jiao Tong University School of Medicine (SH9H-2019-T163-2), and informed consent was obtained from every patient under institutional review board protocols.

### 2.3. Quantitative Real-Time Polymerase Chain Reaction (qRT–PCR)

Total RNA was extracted from cells according to the procedure of RNeasy Kit (Axygen Scientific, CA, USA). The cDNA was reverse-transcribed using a PrimeScript RT Master Mix Kit (Takara, Shiga, Japan). Quantitative PCR was performed on the cDNA using the SYBR Green System (Applied Biosystems, Waltham, MA, USA). GAPDH was amplified used as the endogenous control with commercially available primers (B661104, Sangon Biotech, Shanghai, China). The primers for qRT–PCR are listed in Table S3.

### 2.4. Western Blotting (WB)

Tissues and cells were lysed in RIPA buffer supplemented with protease and phosphatase inhibitors (P0013B, Beyotime, Shanghai, China). Each lysate was separated by 10% SDS polyacrylamide gel electrophoresis and then transferred to Immobilon-P PVDF transfer membranes (Merck Millipore, Burlington, MA, USA). After blocking with 5% non-fat milk, the membranes were incubated with primary antibodies overnight at 4 °C and incubated with HRP–conjugated secondary antibodies at room temperature for 1 h. The protein band signals were detected using an Amersham Imager 600 (General Electric Company, Boston, MA, USA). The antibodies are listed in Table S4.

### 2.5. Cell Transfection

For lentiviral shRNA infection, MPNST cells at 70% confluence were infected with lentivirus vector packaging plasmids (Zorin Biotechnology, Shanghai, China) containing shRNAs targeting NCOR2. A random nonsense targeted sequence was used as the negative control (NC). Cells were infected overnight, and stable cell populations were selected with 2 µg/mL puromycin 48 h later. The target sequence of shNCOR2-A was 5′-GCGGAAGAAGCUAAUCUUGUATT-3′, and that of shNCOR2-B was 5′-CGGAAUGAGCCUGAAUACAAUTT-3′.

### 2.6. Cell Viability Assay

Cell viability was examined using a Cell Counting Kit-8 (CCK-8, Dojindo Laboratories, Kyushu Island, Japan), according to the manufacturer’s instructions. A total of 3 × 10^3^ cells were seeded per well in 96-well plates, and CCK-8 solution was diluted at a ratio of 1:10, and serum-free DMEM was added at 0, 24, 48, and 72 h to measure the 450 nm OD value after 2 h of incubation. 5-Ethynyl-20-deoxyuridine (EdU) assays were performed using an EdU kit (Beyotime, Shanghai, China). Briefly, 5 × 10^3^ cells were seeded per well in 48-well plates. Second day, the cells were incubated with EdU solution for 2 h at 37 °C, fixed with 4% paraformaldehyde for 15 min, permeabilized with 0.3% Triton X-100 (Sigma Aldrich, Saint Louis, MO, USA) for 10 min, and rinsed with phosphate-buffered saline three times. The cells were incubated with Click Reaction Mixture (Beyotime, Shanghai, China) for 30 min at room temperature under protection from light. Nuclei were labelled with Hoechst (Frankfurt am Main, Germany) 33,342 for 5 min.

### 2.7. Colony Formation Assay

A total of 50 cells were evenly seeded in 60 mm plates and cultured for 7 days. The cells were carefully fixed with 4% formaldehyde for 10 min and subsequently washed with PBS before being stained with 1% crystal violet for 5 min. The stain was washed away slowly with phosphate-buffered saline, and the plates were dried at room temperature. Images of the plates were captured, and the colonies were counted using ImageJ 1.51 (National Institutes of Health, Bethesda, MD, USA).

### 2.8. Cell Invasion and Migration Assays

Transwell assays were performed using hanging cell culture inserts (8 µm pore size; Millipore, MA, USA). A total of 5 × 10^4^ cells were seeded on the top compartment, coated with Matrigel (Corning, New York, NY, USA), in 200 μL of serum-free DMEM and gently placed in 24-well plates, to which 600 μL of 15% serum DMEM (Thermo Fisher Scientific, Waltham, MA, USA) had been added in advance. After 16 h of incubation, the invasive cells were stained with 1% crystal violet for 10 min, and the upper cells were gently removed with a cotton swab. Images of three random views were captured, and the cells were counted using ImageJ.

A scratch assay was used to evaluate cell migration ability. Cells were seeded and grown to near-confluence in 6-well plates. A scratch was made along the centerline of each well by using a 200 μL pipette tip. Images of the scratch at different intervals were taken by an inverted microscope. The change in scratch area was measured using ImageJ. Cell mobility = (scratch width at 0 h − scratch width at 24 h)/scratch width at 0 h × 100%.

### 2.9. Immunohistochemistry (IHC)

The sections were deparaffinized, rehydrated through decreasing concentrations of ethanol and were boiled in EDTA buffer (Takara Bio, Shiga, Japan) for 20 min to retrieve the antigenicity. When the buffer had cooled to room temperature, the sections were blocked with 3% H_2_O_2_ for 10 min and incubated with bovine serum albumin for 20 min. The sections were incubated with primary antibodies overnight at 4 °C and with biotin-conjugated secondary antibodies for 20 min at 37 °C. The signal was detected with DAB staining. The antibodies are listed in Table S5.

The IHC of tissue microarray scores were assessed by two independent researchers. In order to eliminate the error caused by different observation conditions, the researcher was asked to finish the assessment of a total microarray within a continuous time interval (about 2 h). When there were different results, a third pathologist would interpret the results again. The proportion of positive cells was scored as follows: negative (−): score 0; < 25% positive cells (+): score 1; 26–50% positive cells (++): score 2; and > 50% positive cells (+++): score 3. Discussion with a third researcher is required if disagreements occur.

### 2.10. TUNEL Assays

An in situ TUNEL cell apoptosis detection kit (Beyotime, Shanghai, China) was used to assess apoptosis in tumour tissues according to the manufacturer’s instructions. The stained sections were visualized under a fluorescence microscope.

### 2.11. Flow Cytometry (FCM) Experiments

For cell cycle analysis, cells were fixed in 70% ethanol 1 day before the experiment, digested with RNaseA, and labelled with propidium iodide. Apoptotic cells were analysed with an Annexin V/FITC kit (BD Biosciences, San Jose, CA, USA) according to the manufacturer’s instructions and analysed by FCM after compound treatment.

### 2.12. RNA Sequencing (RNA-Seq)

Total RNA was isolated using a TRIzol total RNA extraction kit (TIANGEN, Cat. No. DP424, Beijing, China). Illumina HiSeq library construction was performed according to the manufacturer’s instructions (Illumina, San Diego, CA, USA). The library was sequenced using the Illumina NovaSeq 6000 sequencing platform to generate raw reads.

Raw paired-end fastq reads were filtered with TrimGalore (GitHub, San Francisco, CA, USA) and then aligned to the human genome using HISAT2 (Daehwan Kim Lab, Dallas, TX, USA) [29], followed by reference genome-guided transcriptome assembly and gene expression quantification using String Tie (Center for Computational Biology, Baltimore, MD, USA) [30]. Differentially expressed genes (DEGs) were identified by DESeq2 (Bioconductor, Baden-Wuerttemberg, Germany) with cut-off values of a log2|fold-change| > 1 and a *p*-adjust < 0.05. ClusterProfiler (Bioconductor, Baden-Wuerttemberg, Germany) was used to perform functional enrichment analysis and the potential genes in the identified modules were analysed based on gene ontology (GO) and KEGG pathway categories. Each data point is presented as the mean, and all experiments were performed on three biological replicates.

### 2.13. Enzyme-Linked Immunosorbent Assay (ELISA)

A total of 1 × 10^5^ cells were evenly seeded in 60 mm plates and cultured overnight. On the second day, the cells were incubated with serum-free DMEM. The supernatant was collected after 24 h. Peripheral blood of PNF and NF1-derived patients of Shanghai Ninth People’s Hospital (Shanghai, China) was collected. The BDNF levels were detected by the commercially available ELISA kit (Boster Biological Technology Company, Wuhan, China). All experiments were performed in duplicate, and the tests were performed according to the manufacturer’s instructions. 

### 2.14. Xenograft Tumour Models

Sixteen six-week-old female NOD-SCID IL-2 receptor gamma-null mice (purchased from Shanghai Jihui Animals Company, Shanghai, China) were used for xenograft tumour models. A total of 5 × 10^6^ shNCOR2 or shNC cells in 100 μL PBS, containing 50% Matrigel (Corning, New York, NY, USA), were injected subcutaneously into the armpit of each mouse. The maximum allowable volume of tumour growth was approximately 1500 mm^3^. The tumour size and mouse weight were measured twice a week. The volume was calculated as follows: V = L × W^2^/2 (L: length, W: width). All procedures were performed in accordance with the guidelines established by the Shanghai Medical Experimental Animal Care Commission.

### 2.15. Statistical Analysis

Analyses were performed using SPSS Statistics 23.0 (Chicago, IL, USA) and GraphPad Prism version 8.0 (San Diego, CA, USA). Overall survival curves were estimated using the Kaplan–Meier method, and differences in survival were evaluated using the log-rank test. Clinicopathological correlations were analysed by Spearman correlation. All data are shown as mean ± standard deviation (SD) and were analysed by paired or unpaired two-sided *t*-tests. *p* < 0.05 was considered to indicate statistical significance.

## 3. Results

### 3.1. NCOR2 Expression Is Significantly Elevated in NF1-Derived MPNSTs and Is Associated with Patient 10-Year Survival Time

**Figure 1:**
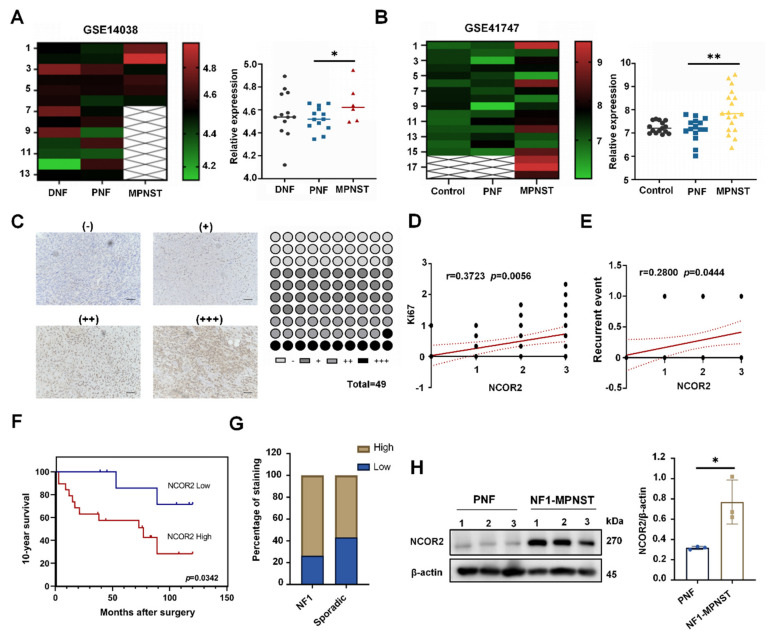
NCOR2 expression is significantly elevated in NF1-derived MPNSTs and is associated with patient 10-year survival time. (**A**) NCOR2 expression was significantly higher in MPNSTs than in PNFs in GSE14038; * *p* < 0.05. (**B**) NCOR2 level was higher in mouse MPNSTs than PNFs in GSE41747; ** *p* < 0.005. (**C**) Representative IHC images of NCOR2 expression in tissue microarray. Scale bar, 50 μm. 63% MPNST tissues showed medium or high NCOR2 expression. (**D**) The expression of NCOR2 was positively correlated with Ki67; r = 0.3723; *p* = 0.0056. (**E**) There is a weakly positive correlation between NCOR2 expression and local recurrence; r = 0.2800; *p* = 0.0444. (**F**) Kaplan–Meier survival analysis showing that the 10-year survival of the NCOR2-high expression group was significantly shorter than that of low expression group, *p* = 0.0342. (**G**) NCOR2 high expression accounted for a larger proportion of NF1-derived MPNSTs (73.7%, 14/19). (**H**) WB showing that NCOR2 protein levels were significantly increased in NF1-derived MPNST fresh samples compared with that in PNFs; β-actin was used as the control. Data are shown as mean ± S.D., *n* = 3; * *p* < 0.05 (seen in Supplementary File S1).

We explored publicly available expression datasets for MPNST patient cohorts, the datasets GSE14038 [31], and GSE41747 [32] from the Gene Expression Omnibus (GEO) Database (Table S6). We found that NCOR2 expression was significantly higher in MPNSTs than in PNFs (*p* < 0.05) in GSE14038. Additionally, in dataset GSE41747, the NCOR2 levels were significantly elevated in mouse MPNSTs (*p* < 0.005) (Figure 1A,B).

Next, we detected the expression of NCOR2 in the human MPNST tissue microarray (16 low-grade; 33 high-grade). Up to 63% of MPNST tissues showed medium or high NCOR2 expression, and 11.1% and 25.9% showed negative and low expression, respectively (Figure 1C). In addition, the correlation between NCOR2 and Ki67, H3K27me3 (marker of PRC2 function) was analysed. The results showed that the NCOR2 and Ki67 were positively correlated (3 < r = 0.3723 < 5, *p* = 0.0056) (Figure 1D), suggesting that NCOR2 may be a marker of proliferation. However, there was a negative correlation (−1 < r = −0.2422 < 0) between NCOR2 and H3K27me3 with no statistical significance (*p* > 0.05) (Figure S1). This result may be affected by the number of tissue samples in MPNST microarray. Moreover, we recorded the initial MPNST as event 0 and the recurrent MPNST as event 1. The correlation analysis between NCOR2 staining score and local recurrence was analysed, and the result showed a weak positive correlation (0 < r = 0.2800 < 3) (Figure 1E). Then, we classified patients into NCOR2-high (2–3 scores) and NCOR2-low (0–1 scores) expression groups. The patient characteristics are shown in Table 1. Kaplan–Meier survival analysis showed that the 10-year survival of the NCOR2-high expression group was significantly shorter than that of the NCOR2-low expression group (*p* = 0.0342, Figure 1F).

Furthermore, we found that NCOR2 high expression accounted for a larger proportion of NF1-derived MPNSTs (73.7%, 14/19). While in sporadic MPNSTs, the proportion of high (56.7%, 17/30) versus low (43.3%, 13/30) NCOR2 expression was essentially equal (Figure 1G), suggesting the protein may play a role in the development of NF1-derived MPNSTs. To further investigate the expression levels of NCOR2 in NF1-derived MPNSTs, we randomly selected three fresh PNF and three NF1-derived MPNST tissues, and the expression level of NCOR2 was analysed by WB. Compared to the PNFs, NCOR2 protein levels were significantly increased in NF1-derived MPNSTs (Figure 1H). The clinical features of the patients are shown in Table S7. These results suggest that NCOR2 may play an important role in the malignant transformation of PNFs into NF1-derived MPNSTs. 

### 3.2. NCOR2 Knockdown Leads to Inhibition of NF1-Derived MPNST Cell Proliferation In Vitro

**Figure 2:**
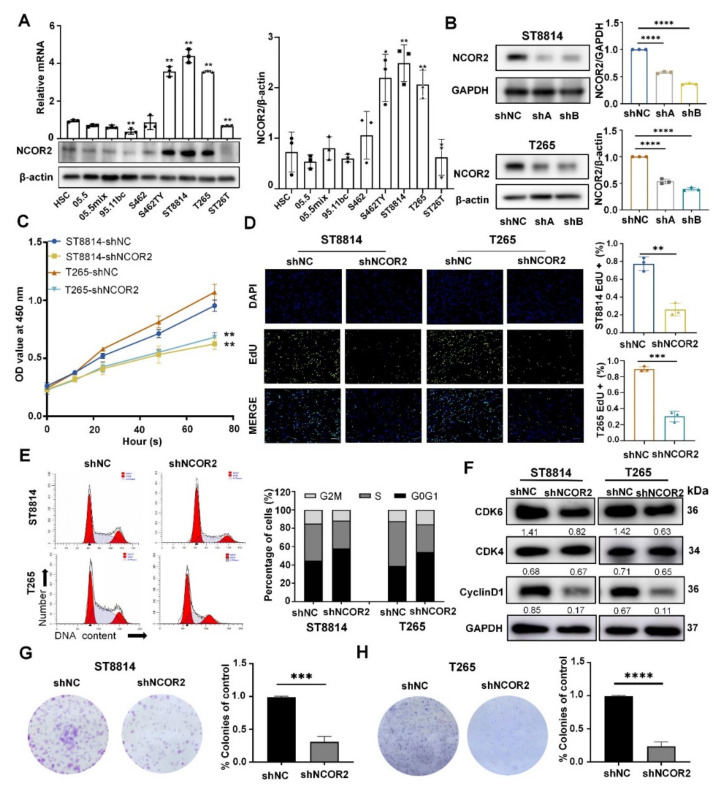
NCOR2 knockdown leads to the inhibition of NF1-derived MPNST cell proliferation in vitro. (**A**) qPCR and WB showing that the NCOR2 mRNA and protein levels in one NHSC, three PNF cell lines, four NF1-derived MPNST cell lines, and one sporadic MPNST cell line; β-actin was used as the control. Data are shown as mean ± S.D., *n* = 3; * *p* < 0.05, ** *p* < 0.005. (**B**) WB showing that the efficacy of NCOR2 knockdown in ST8814 and T265 cells at protein levels; shB was selected for further investigation. GAPDH was used as the control. Data are shown as mean ± S.D., *n* = 3; **** *p* < 0.0001. (**C**,**D**) Significantly decreased cell viability was observed in the shNCOR2 groups by CCK-8 and EdU assays. Data are shown as mean ± S.D., *n* = 3; ** *p* < 0.005, *** *p* < 0.0005. (**E**,**F**) FCM showing that the cells were mostly arrested in G0/G1 phase in the shNCOR2 groups. Data are shown as mean ± S.D., *n* = 3. WB showing that the protein levels of CDK6 and cyclin D1 were decreased, respectively. (**G**,**H**) An evident reduction in the colony formation ability in both ST8814- shNCOR2 and T265- shNCOR2 cell lines. Data are shown as mean ± S.D., *n* = 3; *** *p* < 0.0005; **** *p* < 0.0001.

Next, we further analysed the expression level of NCOR2 in different PNF and MPNST cell lines, including three PNF cell lines, four NF1-derived MPNST cell lines, and one sporadic MPNST cell line by using qPCR and WB (Figure 2A). Compared with other cell lines, the NCOR2 level in three NF1-derived MPNST cell lines (ST8814, T265 and ST462TY) was significantly increased (*p* < 0.005). Therefore, they were selected for later in vitro and in vivo experiments.

In vitro, we transfected ST8814 and T265 cells with lentiviral vectors encoding NCOR2-targeting short hairpin RNAs (shRNAs; shA and shB) or a negative control (shNC) and verified the knockdown efficiency by qPCR and WB (Figure 2B and Figure S2). Through verification, shB was selected for further investigation. According to the CCK-8 and EdU assays, significantly decreased cell viability was observed in the shNCOR2 groups (Figure 2C,D). In addition, FCM experiments were carried out and showed that the cell proliferation cycles were mostly arrested in the G0/G1 phase (Figure 2E). We confirmed that the protein levels of CDK6 and Cyclin D1 were decreased in shNCOR2 group by WB (Figure 2F). In addition, NCOR2 knockdown caused an evident reduction in the colony formation ability in both ST8814 and T265 cell lines (Figure 2G,H). FCM experiments also showed that the shNCOR2 groups displayed a higher apoptosis rate (Figure S3). However, there were no significant changes in cell migration and invasion (Figure S4A,B).

### 3.3. NCOR2 Knockdown Reduces the Tumourigenicity of NF1-Derived MPNST Cells In Vivo

**Figure 3:**
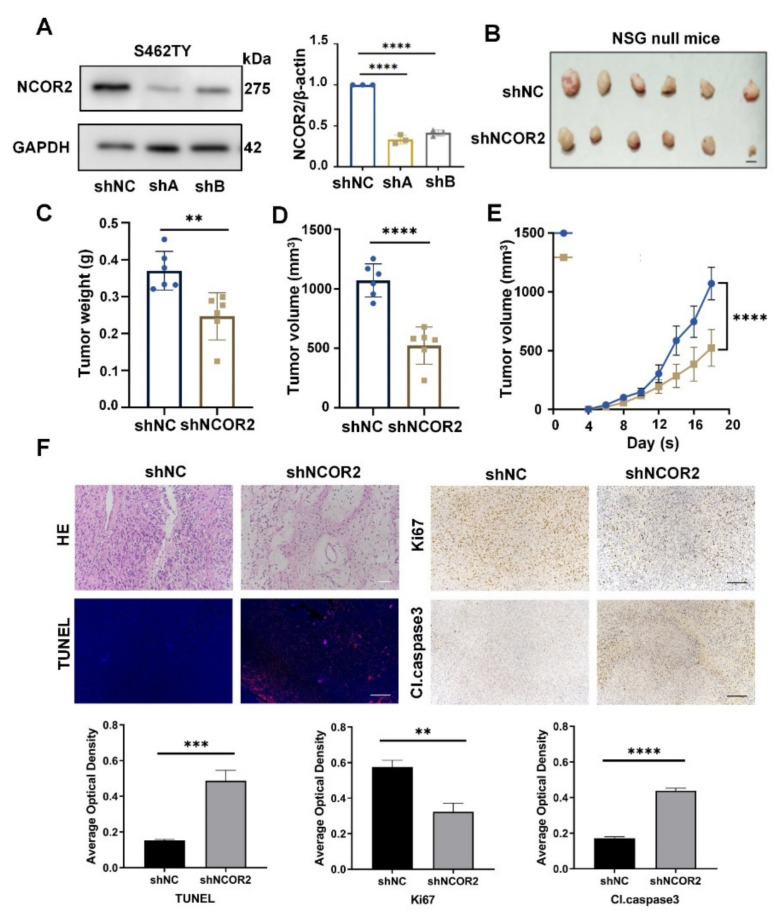
NCOR2 knockdown reduced the tumourigenicity of NF1-derived MPNST cells in vivo. (**A**) WB showing that the efficacy of NCOR2 knockdown in S462TY cells at protein levels; shA was selected for further transplantation. GAPDH was used as the control. Data are shown as mean ± S.D., *n* = 3; **** *p* < 0.0001. (**B**–**E**) The S462TY-shNCOR2 group displayed significant reductions in tumour size and weight. Data are shown as mean ± S.D., *n* = 6; ** *p* < 0.005, **** *p* < 0.0001. (**F**) HE and IHC showing that the density of tumour cells was reduced, the positive expression of Ki67 was decreased and cleaved-caspase 3 was increased in S462TY-shNCOR2 group; TUNEL staining showing increased apoptotic cells in S462TY-shNCOR2 group. Scale bar, 50 μm. Data are shown as mean ± S.D., *n* = 3; ** *p* < 0.005, *** *p* < 0.0005, **** *p* < 0.0001.

To further verify the roles of NCOR2 in vivo, we constructed xenograft models. We transfected ST462TY cells with shNC and shNCOR2s and verified the knockdown efficiency by WB (Figure 3A). T265 and ST8814 were not used due to their low tumourigenicity, as previously described [28,33]. Then, we selected the shA group for the following transplantation. Compared with the control group, the shNCOR2 group displayed significant reductions in tumour size and weight (Figure 3B–E). There was no significant difference in the body weights of the mice (Figure S5). Then, the xenograft tumours were collected, made into paraffin sections, and evaluated by haematoxylin and eosin (HE) staining and IHC. In the shNCOR2 group, the density of tumour cells was reduced, the positive expression of Ki67 was decreased, and cleaved caspase 3 was increased. In addition, TUNEL staining also demonstrated increased apoptotic cells in tumour tissues (Figure 3F).

### 3.4. NCOR2 Regulates the MAPK Signalling Activation in NF1-Derived MPNST Cells

**Figure 4:**
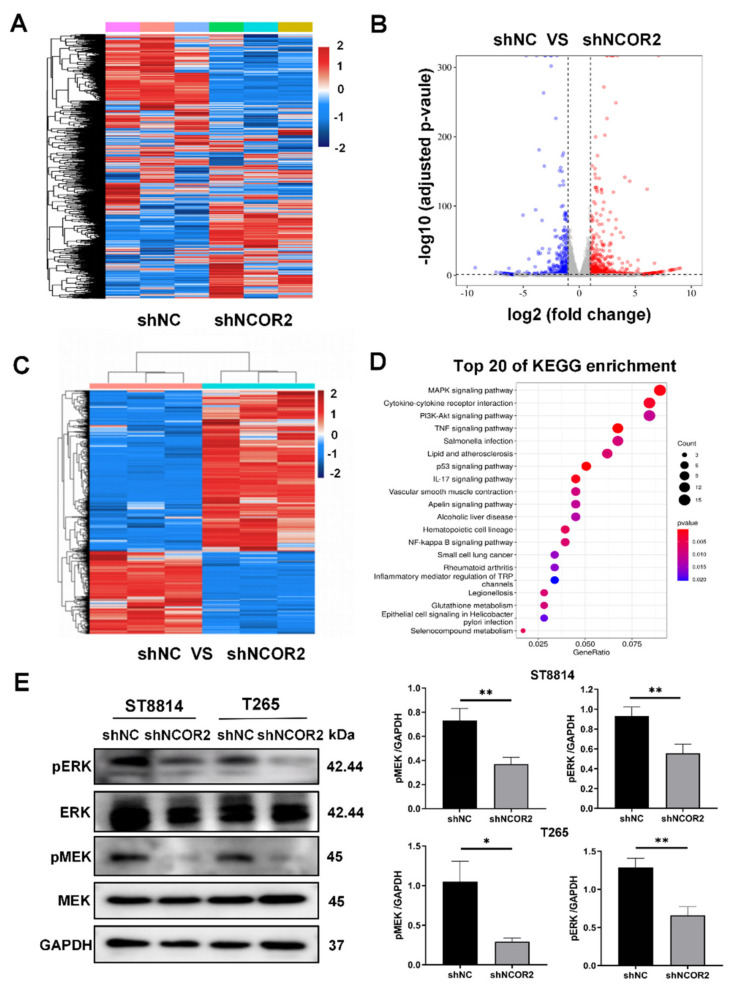
NCOR2 regulates the MAPK signalling activation in NF1-derived MPNST cells. (**A**) The total RNA was extracted from T265-shNCOR2 and shNC cells, and RNA-Seq profiling was performed. (**B**) Volcano plot of RNA-Seq results. Red plots represent significantly upregulated proteins, whereas blue plots represent significantly downregulated proteins in T265-shNCOR2 cells. Dashed lines indicate the significance threshold; *p*-adjust < 0.05, |Log FC| > 1. (**C**) Clustering analysis of significantly different expressed proteins. Blue, low expression; red, high expression; *n* = 6. (**D**) KEGG pathway enrichment analyses showing that attenuated activation of several tumourigenic pathways. (**E**) WB showing that NCOR2 knockdown altered the phosphorylation level of ERK1/2 and MEK1/2 in ST8814 and T265 cells. * *p* < 0.05, ** *p* < 0.005.

Subsequently, we explored the mechanisms underlying the roles of NCOR2 in NF1-derived MPNST cells. We extracted total RNA from T265 cells transfected with shNC and shNCOR2 and performed RNA-Seq profiling (Figure 4A). Three pairs of samples were tested to ensure the stability of the results (GSE201668). According to the data of differential expression analysis using DESeq2, the number of upregulated genes (919) was higher than the number of downregulated genes (469). These genes had at least a two-fold expression change (*p* < 0.05) following NCOR2 knockdown (Figure 4B,C).

According to the KEGG pathway analyses, we observed attenuated activation of several tumourigenic pathways, and the most significantly enriched pathway for differentially expressed genes was the MAPK pathway (Figure 4D). We verified the expression of the top 10 downregulated genes in this signalling pathway by qPCR, and the change trend matched the results of RNA-Seq (Figure S6). Then, the activation of MEK and ERK was verified by WB and IHC (Figure 4E and Figure S7), and we can see decreased expression of phosphorylated MEK and ERK proteins. In short, these data suggest that downregulation of NCOR2 suppresses MAPK signalling activation.

### 3.5. BDNF Plays a Key Role in ERK Signal in NF1-Derived MPNST Cells

**Figure 5:**
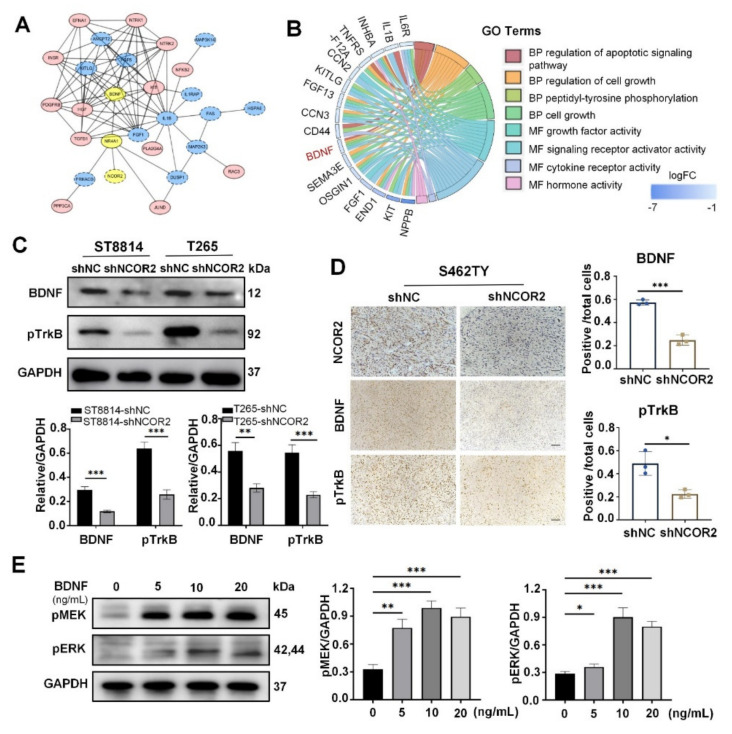
BDNF can regulate the MAPK signalling pathway in NF1-derived MPNST cells. (**A**) Protein–protein interaction network showing that BDNF occupied a central position among the altered genes in MAPK signalling pathway. (**B**) GO enrichment analyses showing BDNF plays a role in related cell growth and apoptosis processes. (**C**,**D**) WB and IHC showing BDNF and pTrkB protein levels were decreased in ST8814-shNCOR2 and T265-shNCOR2 cells. Scale bar, 50 μm. Data are shown as mean ± S.D., *n* = 3; * *p* < 0.05, ** *p* < 0.005, *** *p* < 0.0005. (**E**) T265-shNCOR2 was treated with human recombinant BDNF at 5 ng/mL, 10 ng/mL, and 20 ng/mL. After 24 h, WB showed pMEK and pERK expression evidently increased. * *p* < 0.05, ** *p* < 0.005, *** *p* < 0.0005.

To further investigate the substrate of NCOR2 in NF1-derived MPNST cells, we first used the STRING database [34] and cystoscope to construct a protein–protein interaction network. The results showed that brain-derived neurotrophic factor (BDNF) occupied a central position among the altered genes in the MAPK signalling pathway (Figure 5A). Then, via GO analyses, we observed that BDNF plays a role in related cell growth and apoptosis processes (Figure 5B). Due to BDNF being a secretory protein, the supernatants of normal human Schwann cell, PNF cells, NF1-derived MPNST cells, and sporadic MPNST cell without knockdown of NCOR2, and the peripheral blood of 3 PNF patients and NF1-derived MPNST patients, were collected to detect the content of BDNF through ELISA. We can see that the content of BDNF secreted by NF1-derived MPNSTs is significantly higher than that of other cell lines. The BDNF content in the blood samples of NF1-derived MPNST patients was significantly higher than that of PNF patients, indicating that BDNF plays a role in the development of NF1-derived MPNSTs (Figure S9). Next, we verified that BDNF and pTrkB protein levels were decreased in shNCOR2 groups by WB and IHC, suggesting that there is a regulatory role between the two proteins. (Figure 5C,D).

Previous studies have demonstrated that BDNF/TrkB signal can directly promote ERK cascade activation [35,36,37,38]. Therefore, we speculated that NCOR2 regulated the activation of the ERK pathway via BDNF/TrkB signal. To test this hypothesis, we treated T265-shNCOR2 cells with human recombinant BDNF at 5 ng/mL, 10 ng/mL, and 20 ng/mL. After 24 h, the WB results showed that pMEK and pERK expression was significantly increased (Figure 5E). The above results indicate that NCOR2 regulates MAPK signalling pathway maybe through regulating the expression level of BDNF in NF1-derived MPNST cells. 

### 3.6. Human Recombinant BDNF Can Reverse the Effects of NCOR2 Knockdown on NF1-Derived MPNST Cells

**Figure 6:**
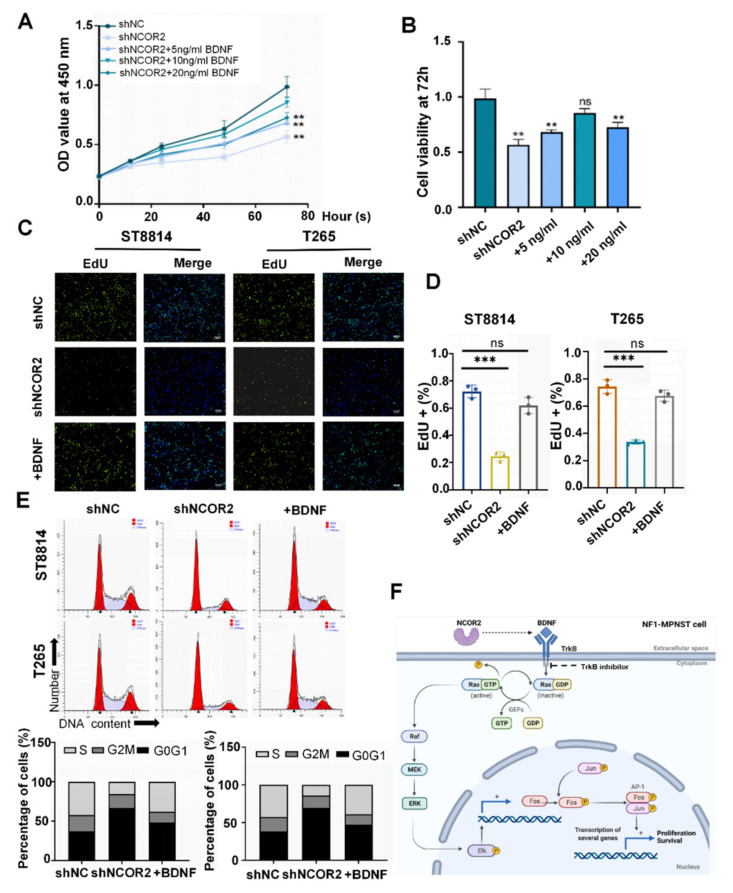
Human recombinant BDNF can reverse the effects of NCOR2 knockdown on NF1-derived MPNST cells. (**A**,**B**) CCK-8 assays showing the growth of all T265-shNCOR2 groups was promoted, and the effect with 10 ng/mL human recombinant BDNF was the most obvious, ** *p* < 0.05. (**C**,**D**) EdU assays showing that ST8814-shNCOR2 and T265-shNCOR2 cells with 10 ng/mL human recombinant BDNF had a higher growth rate than those without BDNF. Data are shown as mean ± S.D., *n* = 3; ns, not significant; *** *p* < 0.0005. (**E**) The cell cycle assays showing the proportion of proliferative cells increased evidently after ST8814-shNCOR2 and T265-shNCOR cells treated with human recombinant BDNF. (**F**) The BDNF protein in NF1-derived MPNST cells plays a key role in NCOR2-mediated MAPK activation.

To further clarify the biological role of BDNF in shNCOR2 NF1-derived MPNST cells, we performed CCK-8 assays when the T265-shNCOR2 cells were treated with human recombinant BDNF at 5 ng/mL, 10 ng/mL, and 20 ng/mL. We observed that the growth of all groups was promoted, and the effect with 10 ng/mL was the most obvious (Figure 6A,B). Therefore, this condition was selected for the following study. In EdU assays, we observed that NCOR2-knockdown ST8814 and T265 cells with BDNF had a higher growth rate (Figure 6C,D). Moreover, we detected the cell cycle and apoptosis rate, and the results showed that the cell proportion of S phase increased, while that of apoptotic cells decreased (Figure 6E and Figure S8). To a large extent, human recombinant BDNF reversed the effects of NCOR2 knockdown on NF1-derived MPNST cells.

In short, NCOR2 knockdown can inhibit NF1-derived MPNST cell proliferation by weakened BDNF/TrkB/ERK signalling (Figure 6F).

## 4. Discussion

MPNSTs are markedly aggressive and chemoresistant peripheral soft-tissue sarcomas. In NF1 patients, a rapid change in size or the onset of pain from PNF may suggest malignant transformation to MPNST. Clinically, NF1-derived MPNSTs are often diagnosed at an advanced stage, with primary tumours having reached a diameter of 5–10 cm and even having broken through the sheath membrane with detectable distant metastases [39]. Recent years have witnessed significant interest in signal transduction pathways occurring in these tumours [40,41,42], so many investigations have developed a few drugs targeting intermediates that promote proliferation in pro-oncogenic pathways [43,44,45,46,47]. However, most drugs have yet to show definite efficacy in clinical trials. This may be because other driver mutations remain unknown, so efforts should be made to obtain new insights into the growth mechanism of MPNSTs and to identify novel therapeutic targets.

Compelling evidence has been reported that transcriptional corepressors play crucial roles in phylogeny and tumour progression [15]. Among them, altered NCOR2 expression has been reported and investigated in various tumours [19,20,21,48]. The inhibitory or oncogenic effects of NCOR2 show heterogeneity among different types of cancer [21,24,48,49]. Moreover, by using the Gene Expression Profiling Interactive Analysis Database, we found that the correlation between high NCOR2 levels and overall survival time was different in different tumours. For example, in cervical squamous cell carcinoma and endocervical adenocarcinoma and ovarian serous cystadenocarcinoma, patients with elevated NCOR2 levels had shorter overall survival time, but in adrenocortical carcinoma and bladder urothelial carcinoma, there was no correlation between the NCOR2 level and overall survival (Figure S10A–D). However, to date, little is known about the function and underlying mechanism of NCOR2 in MPNSTs. In this study, MPNST patients with higher NCOR2 levels had shorter 10-year survival times, suggesting that NCOR2 is associated with poor prognosis in MPNSTs. Moreover, we found, both in tissues and cell lines, that elevated NCOR2 expression is more closely related to NF1-derived MPNSTs. Therefore, we performed cell transfection coupled with xenograft studies and transcriptomic sequencing to explore the biological roles of NCOR2 in NF1-derived MPNSTs. By carrying out experiments in vitro and in vivo, we found that decreased NCOR2 expression inhibited NF1-derived MPNST cell proliferation by weakening BDNF/TrkB/ERK signalling.

BDNF is a growth factor belonging to the neurotrophic family and plays critical roles in regulating neuron differentiation, survival, synaptic function, and plasticity in the nervous system [50,51,52]. According to the protein–protein interaction network, NCOR2 has no direct interaction with BDNF, and they are related through nuclear receiver subfamily 4 group A member 1 (NR4A1). NR4A1 expression was up-regulated in MAPK signaling pathway of RNA-seq. Previous studies have shown that NR4A1 can play a role as a tumour suppressor in association with BNDF [53]. Therefore, it can be speculated that NCOR2 may affect the biological function of tumour cells by regulating the expression of BDNF through NR4A1. TrkB is the specific binding receptor of BDNF, a member of the neurotrophic receptor tyrosine kinase family [54]. BDNF, as a non-covalently linked homologous dimethyl protein, will trigger the TrkB receptor dimerization when it binds to the receptor, and the receptor dimerization will induce autophosphorylation of the kinase domain in the cytoplasm of its own and then activate the downstream signal pathway [55]. Studies have demonstrated that BDNF/TrkB can activate the Ras/MEK/ERK and PI3K/AKT pathways for neuronal survival [56]. Abnormal BDNF/TrkB signal increases the possibility of tumour formation in the nervous system, such as neuroblastomas, glioma, and medulloblastomas [57,58,59] (Table S8). In addition, studies have indicated that BDNF activation of TrkB can induce chemoresistance through activation of the PI3K/AKT and MAPK pathways in neuroblastomas [36,60,61,62]. Notably, TrkB fusions have often been found in nerve-derived tumours (Table S9). These fusion proteins promote cell proliferation and survival by activating downstream oncogenic signalling pathways. Therefore, whether TrkB fusion exists in MPNSTs needs further discussion in the future. To alleviate these abnormalities, several small-molecule compounds targeting TRK fusions have been developed and tested in preclinical and clinical trials, and they have shown remarkable antitumour activities in different cancers, including different neurogenic neoplasms [63,64]. This study firstly suggests that further developing inhibitors targeting BDNF/TrkB signalling has great application prospects in the treatment of NF1-derived MPNSTs.

## 5. Conclusions

In summary, our findings demonstrated that NCOR2 expression is significantly elevated in NF1-derived MPNSTs and is associated with patient 10-year survival time. NCOR2 knockdown results in inhibition of NF1-derived MPNST cell proliferation and increased cell apoptosis in vitro and vivo by weakened BDNF/TrkB/ERK signalling. To further develop inhibitors targeting BDNF/TrkB signalling in NF1-derived MPNSTs alone or in combination with ERK inhibitors may be a novel therapeutic strategy for clinical trials.

## Figures and Tables

**Table 1:** Clinical parameter with NCOR2 expression.

	NCOR2 Expression		*p*-Value
	high	low	
Gender			
Male	15	11	0.449
Female	19	9	
Age			
<45	18	10	0.838
>45	16	10	
Tumor size			
T1 (<5)	9	8	0.447
T2 (5–10)	10	5	
T3 (10–15)	6	4	
T4 (>15)	5	2	
Tumor site			
Head and neck	11	4	0.292
Trunk	9	6	
Limbs	11	9	
NF1			
With	14	6	0.427
Without	17	12	

## Data Availability

RNA-Seq datasets supporting the conclusions of this article are stored in the GEO database (https://www.ncbi.nlm.nih.gov/geo/query/acc.cgi?acc=GSE201668 (1 July 2023)). The data presented in this study are available in the article and in supplementary material here.

## Supplementary Materials

The following supporting information can be downloaded at: https://www.mdpi.com/article/10.3390/cancers14235798/s1.
