# Supplementary material for: Knockdown of NCOR2 Inhibits Cell Proliferation via BDNF/TrkB/ERK in NF1-Derived MPNSTs
Source: Cancers (Basel). 2022 Nov 24;14(23):5798. doi: 10.3390/cancers14235798 (PMC9738545; doi:10.3390/cancers14235798)
Supplement: Supplementary file 1 [file cancers-14-05798-s001.zip › Supplementary figures and tables.pdf]

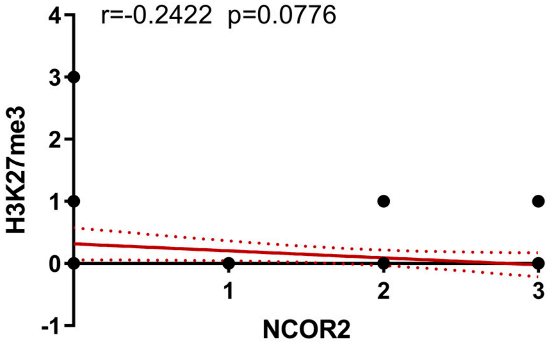

Figure S1 The correlation between NCOR2 and H3K27me3.The results show that there is no correlation between NCOR2 and H3K27me3 (P=0.8472).

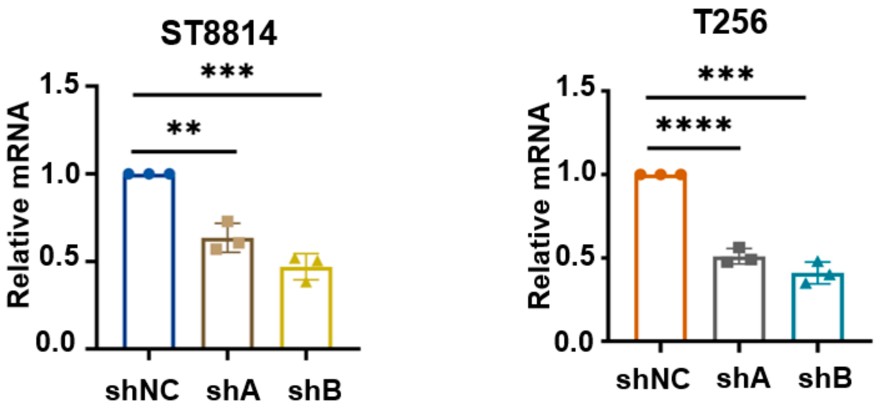

Figure S2 qPCR showing that the efficacy of NCOR2 knockdown in ST8814 and T265 cells at mRNA levels; shB was selected for further investigation. Data were shown as the mean ± S.D., n = 3; **P<0.005; ***P<0.0005; ****P<0.0001.

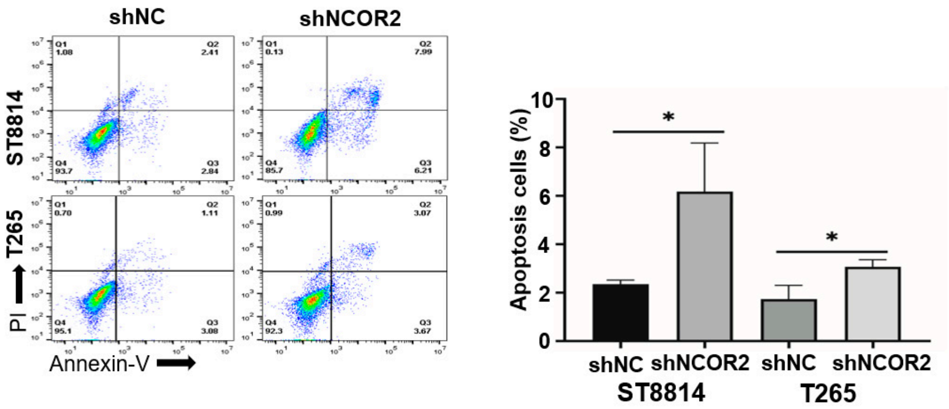

Figure S3 FCM also showed that the shNCOR2 groups displayed a higher apoptosis rate. Data were shown as the mean ± S.D., n = 3; *P<0.05.

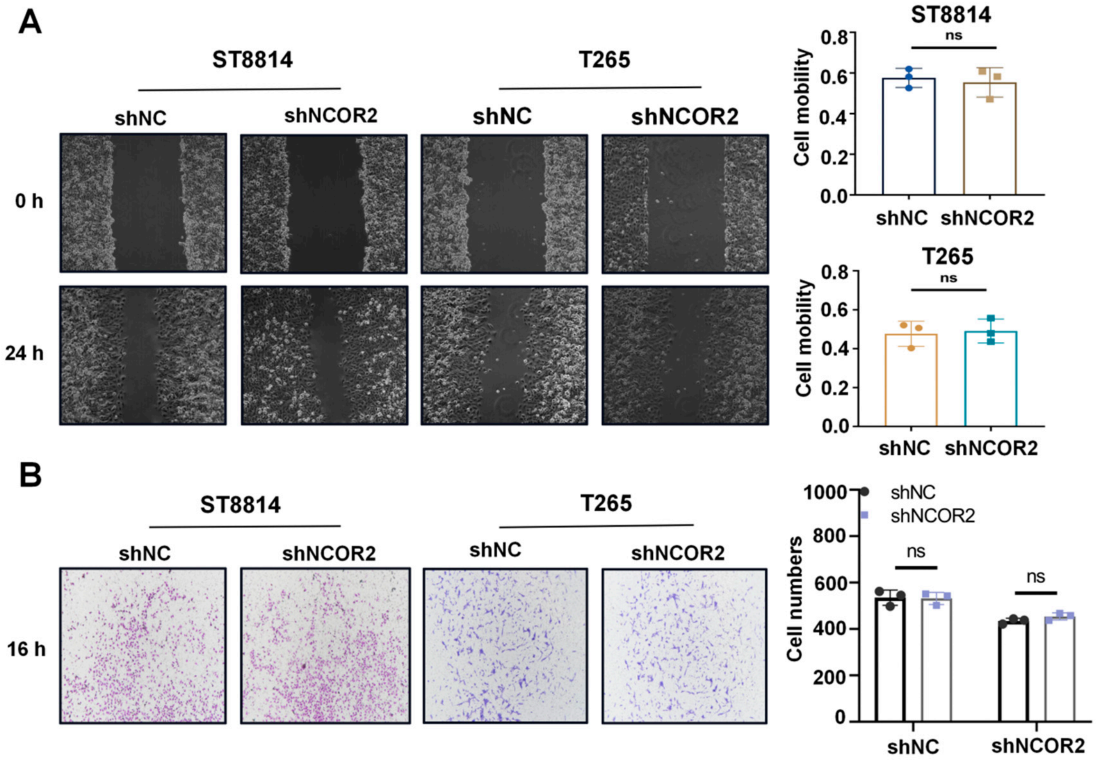

Figure S4 NCOR2 knockdown does not affect the cell migration and invasion ability in NF1-derived MPNST cells. (A) Scratch assays showing that NCOR2 knockdown did not suppress ST8814 and T265 cell migration into the scratching area. Data were shown as the mean ± S.D., n = 3; ns, not significant. (B) Matrigel-transwell invasion assay showing the invasive capability of NCOR2-knockdown ST8814 and T265 cells did not altered. Data were shown as the mean ± S.D., n = 3; ns, not significant.

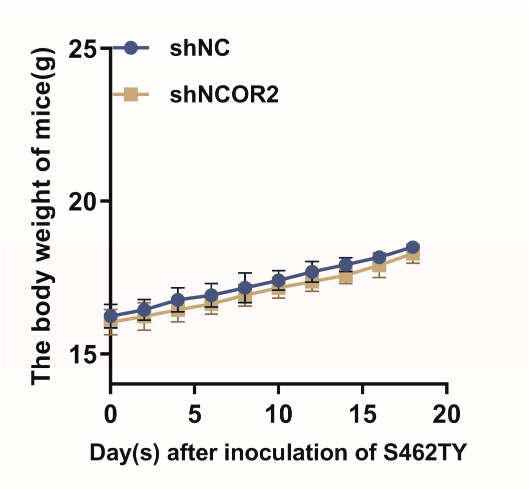

Figure S5 There was no significant difference in the body weights of the mice. shNC, n=6; shNCOR2, n=6.

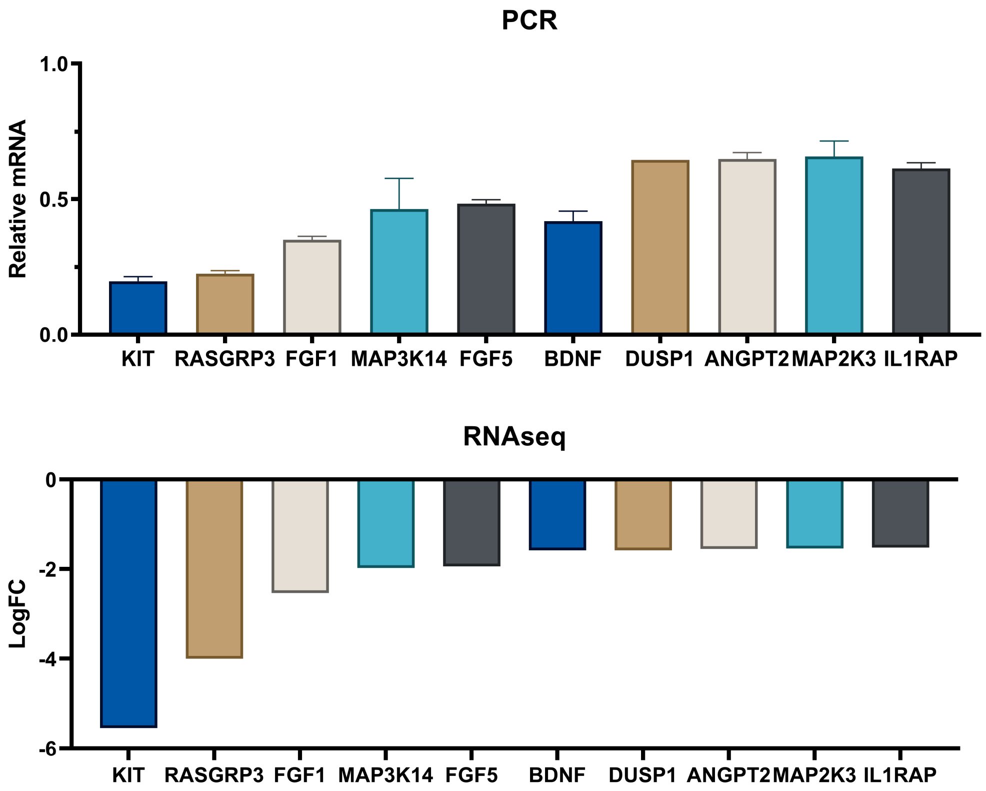

Figure S6 qPCR showing that the change trend of top 10 downregulated genes expression in MAPK signalling pathway matched the RNA-Seq results.

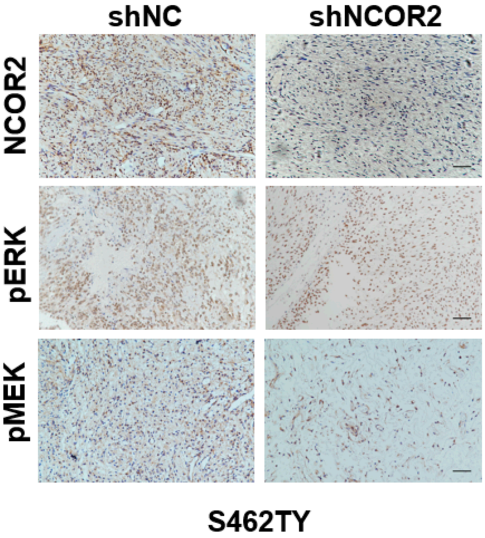

Figure S7 IHC showing the positive expression of pERK and pMEK were decreased in NCOR2-knockdown S462TY cells. Scale bar, 50 μM.

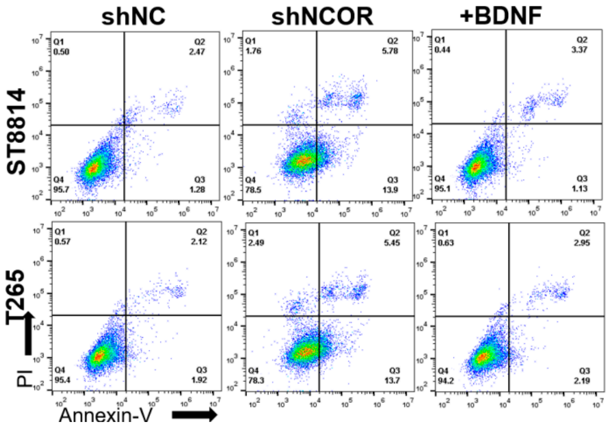

Figure S8 FCM showing that the proportion of apoptotic cells decreased evidently after the ST8814-shNCOR2 and T265-shNCOR2 treated with human recombinant BDNF.

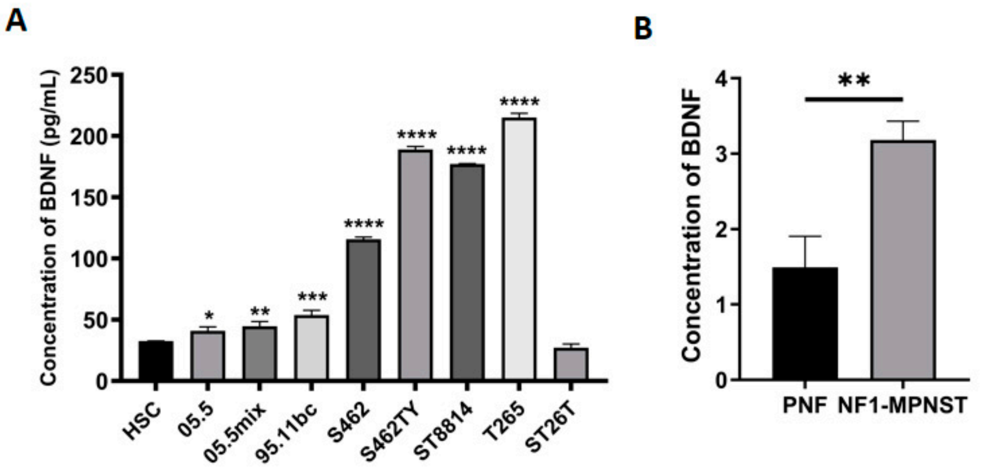

Figure S9 BDNF content in cell supernatant and peripheral blood of patients. (A) ELISA showing that the content of BDNF secreted by NF1-derived MPNSTs (S462, S462TY, ST8814, T265) is significantly higher than that of other cell lines, especially those with high NCOR2 level. (B) ELISA showing that the content of BDNF in peripheral blood from NF1-derived MPNST patients was significantly higher than those from PNF patients. Data were shown as the mean ± S.D., n = 3; **P<0.005.

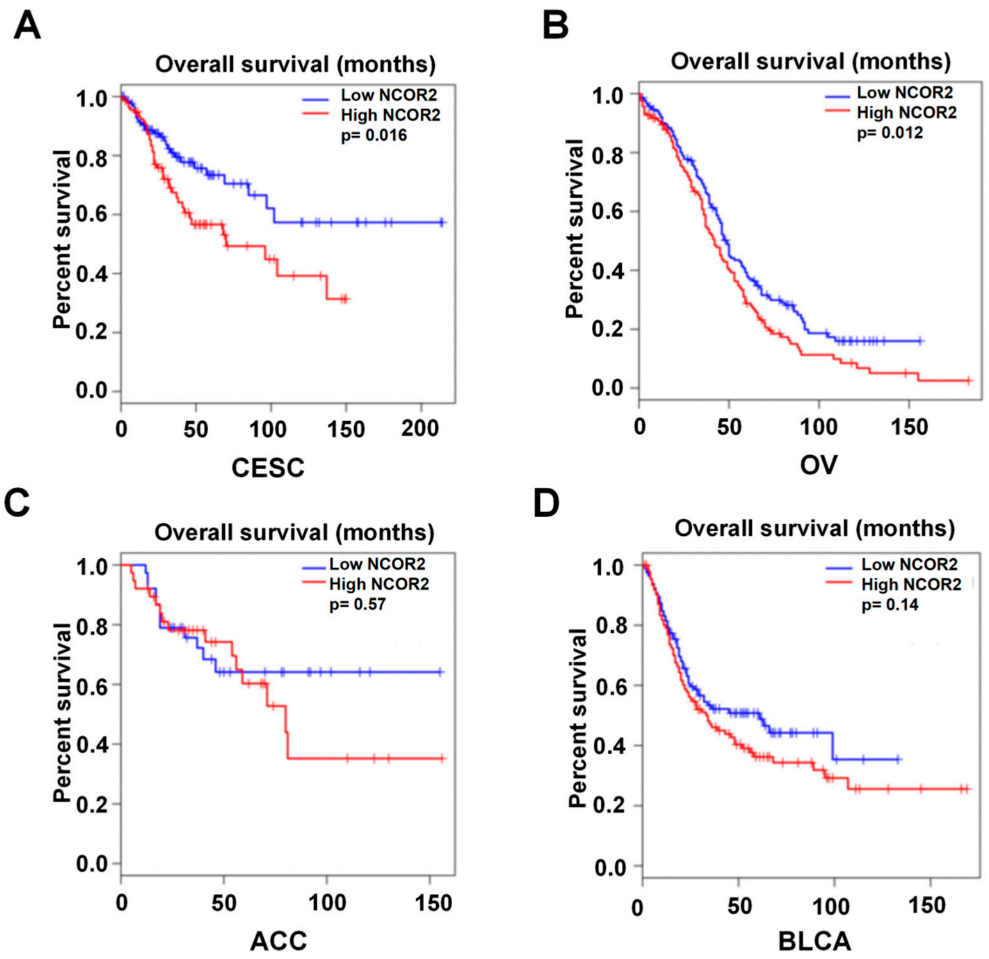

Figure S10 The correlation between high NCOR2 levels and overall survival time was different in other tumours. (A, B) patients with elevated NCOR2 levels had shorter overall survival times in CESC and OV. (C, D) There was no correlation between the NCOR2 level and overall survival in ACC and BLCA. ACC: adrenocortical carcinoma; BLCA: bladder urothelial carcinoma; CESC: cervical squamous cell carcinoma and endocervical adenocarcinoma.

Table S1. The differential diagnosis of NF1-associated nerve sheath tumours

PNF	Neurofibroma diffusely enlarging and replacing a nerve, often involving multiple nerve fascicles, delineated by EMA+ perineurial cells
ANNUBP	Schwann cell neoplasm with at least 2 of 4 features: cytologic atypia, loss of neurofibroma architecture, hypercellularity, mitotic index 1/50 HPFs and 3/10 HPFs
MPNST, low-grade	Features of ANNUBP, but with mitotic index of 3-9/10 HPFs and no necrosis
MPNST, High-grade	MPNST at least with mitotic index of 10/10 HPFs or 3–9/10 HPFs combined with necrosis

PNF: plexiform neurofibroma; ANNUBP: atypical neurofibromatous tumour of uncertain; MPNST: malignant peripheral nerve sheath tumour; HPF:high-power field.

Table S2. FNCLCC Histological Grading System

**tumour Differentiation**	**Score**
Sarcoma closely resembling normal adult mesenchymal tissue	1
Sarcomas for which histologic typing is certain	2
Embryonal and undifferentiated sarcomas, sarcomas of doubtful type, and synovial sarcomas	3
**Mitotic Count**	**Score**
0–9 mitoses per 10 HPF	1
10–19 mitoses per 10 HPF	2
≥20 mitoses per 10 HPF	3
**tumour Necrosis**	**Score**
No necrosis	0
<50% tumour necrosis	1
≥50% tumour necrosis	2

FNCLCC: Fédération Nationale des Centres de Lutte Contre Le Cancer; HPF: high-power field.

Table S3 Sequences of primers used in quantitative RT-PCR

Target Gene	Primer	Nucleotide sequence (5' to 3')
NCOR2	F	GAGGTGTCAGAGATCATCGATG
	R	TCATGTTGATGAACTTGATGCG
KIT	F	CTACAGCCTTATGACGAAATGC
	R	CTTCTCTTCCTCCAGGATTGTG

RASGRP3	F	GATGAGGTGTTTGAGTTCCCT
	R	CTGTAGCCTCACAGAGATCTTG
FGF1	F	CACAGACACCAAATGAGGAATG
	R	CATTCTTCTTGAGGCCAACAAA
MAP3K14	F	GAATTGTCCCTTTGTATGGAGC
	R	ATCCTTCGTGAGTGGAGGTATT
FGF5	F	CCCACGAAGCCAATATGTTAAG
	R	CACTTGCATGGAGTTTTCCTTT
BDNF	F	GACACTTTCGAACACGTGATAG
	R	TACAAGTCTGCGTCCTTATTGT
DUSP1	F	AGGCCATTGACTTCATAGACTC
	R	AGTCCTCATAAGGTAAGCAAGG
ANGPT2	F	CAACACTCAGTGGCTAATGAAG
	R	GCATTCTGCTGTATCTCTACCA
MAP2K3	F	ATCTACAGCACCGACTATTACC
	R	GGTGAACTTACGGTACAGGATG
IL1RAP	F	CAGAAAATGGACGTACGTTTCA
	R	TAGACCGTACAGGGAATGAGTA

Table S4 Antibodies used for the western blot experiments.

Antigen	Dilution	Catalogue	Source
GAPDH	1:5000	#2118	CST
β-actin	1:5000	#3700	CST
NCOR2	1:500	D161011	Sangon Biotech
MEK	1:1000	#4694	CST
pMEK	1:1000	#9154	CST
ERK	1:1000	#4696	CST
pERK	1:1000	#4370	CST
AKT	1:1000	#4691	CST
pAKT	1:1000	#4060	CST
Caspase-3	1:1000	#9662	CST
Cleaved caspase-3	1:1000	#9661	CST
CDK4	1:1000	#12790	CST
CDK6	1:1000	#13331	CST
Cyclin D1	1:1000	#55506	CST
BDNF	1:1000	ab108319	Abcam
pTrkB	1:1000	ab134155	Abcam
anti-mouse (Secondary antibody)	1:5000	ab6728	Abcam
Anti-rabbit (Secondary antibody)	1:5000	Ab6721	Abcam

CST: Cell Signaling Technology, Massachusetts, US; Sangon Biotech: Shanghai, China; Abcam: Burlingame, California, US.

Table S5 Antibodies used for the immunofluorescence experiments.

Antigen	Dilution	Catalog	Source
NCOR2	1:50	D161011	Sangon Biotech
Ki67	1:200	#9449	CST
H3K27me3	1:100	ab6002	Abcam
Cleaved caspase-3	1:400	#9661	CST
pERK	1:200	#4370	CST
BDNF	1:100	ab108319	Abcam
pTrkB	1:100	ab134155	Abcam

CST: Cell Signaling Technology, Massachusetts, US; Sangon Biotech: Shanghai, China; Abcam: Burlingame, California, US.

Table S6 The information of GEO datasets used in this study.

	GSE14038	GSE41747
Website	https://www.ncbi.nlm.nih.gov/geo/query/acc.cgi?acc=GSE14038	https://www.ncbi.nlm.nih.gov/geo/query/acc.cgi?acc=GSE41747
Organism	Homo sapiens	Mouse/Homo sapiens
Country	USA	USA
Platform	GPL7869	GPL10371/GPL14757
Patient population	32 patients	83 patients
Data processing	Microarrays were analyzed with Affymetrix Microarray Suite 5.0 to generate “CEL” files that were processed using Robust Multichip Analysis (RMA) in Bioconductor/R. A custom CDF was used (Brainarray, Hs133P_Hs_REFSEQ_8).	Microarrays were analyzed with Affymetrix Microarray Suite 5.0 to generate “CEL” files that were processed using Robust Multichip Analysis (RMA) in Bioconductor/R. A custom CDF was used for each species (mouse: Brainarray, Mouse4302_Mm_REFSEQ_11.0.1; Human, Brainarray, HGU133Plus2_Hs_REFSEQ_11.0.1).
Age, Sex, Stage, Grade	https://www.ncbi.nlm.nih.gov/geo/query/acc.cgi?acc=GSE14038	https://www.ncbi.nlm.nih.gov/geo/query/acc.cgi?acc=GSE28245

Table S7 Clinical features of fresh NF1-derived MPNST tissue samples

Patients			Histological Findings		Clinical Features				
ID	Gender	Age	H&E	S100	CALMs	CNF	PNF	MPNST	Family History
P1	Female	9	Benign	+	Y	N	Y	N	Y
P2	male	19	Benign	+	Y	N	Y	N	N
P3	male	29	Benign	+	Y	N	Y	Y	N
P4	Female	29	Malignant	+	Y	Y	N	Y	N
P5	Female	/	Malignant	+	Y	N	Y	Y	/
P6	male	29	Malignant	+	Y	N	Y	Y	N

Table S8 Abnormal BDNF/TrkB signal in the nerve-derived tumours

Year	Author	Signal	tumour Type	biomedical functional impacts
1995	Matsumoto K et al.	BDNF and TrkB overexpressed	Neuroblastoma	Cell survival, differentiation, invasiveness
1998	Tajima Y et al.	BDNF and TrkB overexpressed	Medulloblastomas, glial tumours, ependymal tumours	Neuronal survival
1999	Encinas M et al.	BDNF/TrkB/Ras/MAPK	Neuroblastoma	Neuronal differentiation
1999	Encinas M et al.	BDNF/TrkB/PI3K/AKT	Neuroblastoma	Neuronal survival
2002	Ho R et al.	BDNF/TrkB/PI3K/AKT	Neuroblastoma	Drug resistance
2002	Jaboin J et al.	BDNF/TrkB/PI3K	Neuroblastoma	Drug resistance
2005	LI Z et al.	BDNF/TrkB/PI3K/AKT	Neuroblastoma	Drug resistance
2006	Nakamura K et al.	BDNF/TrkB/PI3K/mTOR/VEGF	Neuroblastoma	Angiogenesis
2015	Xiong J et al.	BDNF and TrkB overexpressed	Glioma	Positively associated with malignancy grade
2016	Hua Z et al.	BDNF/TrkB/PI3K/mTOR	Neuroblastoma	Cell metastasis
2019	Wang X et al.	BDNF/TrkB/VGF	Glioblastoma	Cell growth，cell survival and self-renewal

Table S9 TrkB fusions in the nerve-derived tumours

Year	Author	Fusion Protein	Chromosomal Location	tumour Type
2013	Jones, D. T et al.	NACC2-NTKR2	9q34.3	Pilocytic astrocytoma pilocytic
2013	Jones, D. T et al.	QKI-NTKR2	6q26	Astrocytoma
2013	Frattini, V et al.	QKI-NTKR2	6q26	Glioblastomas
2013	Zhang, J et al.	NAVI-NTKR2	1q32.1	Dysembryoplastic neuroepithelial tumours
2014	Stransky, N et al.	AFAP1-NTKR2	4p16.1	Low-grade glioma
2014	Wu, G et al.	QKI-NTKR2	6q26	high-grade Glioma
2014	Stransky, N et al.	AGBL4-NTKR2	1p33	Glioblastomas
2014	Stransky, N et al.	VCL-NTKR2	10q22.2	Glioblastomas
2014	Stransky, N et al.	PML-NTKR2	15q24.1	Pilocytic astrocytoma
2016	Qaddoumi, I et al.	SL-MAP NTRK2	3p14.3	Gangliogliomas
2017	López, G. Y et al.	KANK1-NTKR2	9p24.3	Pilocytic astrocytoma
2018	Prabhakaran, N et al.	TLE4-NTKR2	9q21.31	Gangliogliomas
2019	Jones, K et al.	BCR-NTKR2	22q11.23	Low-grade glioma
2020	Khuong-Quang DA et al.	SPECC1L-NTRK2	22q12.15	Low-grade neuroepithelial tumour
2020	Khuong-Quang DA et al.	SPECC1L-NTRK2	22q11.15	Anaplastic astrocytoma
2020	Pattwell SS et al.	PML-NTRK2	15q24.1	pilocytic astrocytoma
2021	Gupte A et al.	BCR-NTKR2	22q11.23	spinal gangliocytoma
2021	Deland L et al.	GKAP1-NTRK2	9q21.32	low-grade glioma
