# Supplementary material for: Knockdown of NCOR2 Inhibits Cell Proliferation via BDNF/TrkB/ERK in NF1-Derived MPNSTs
Source: Cancers (Basel). 2022 Nov 24;14(23):5798. doi: 10.3390/cancers14235798 (PMC9738545; doi:10.3390/cancers14235798)

## Supplementary file 1

**Figure 1H**

**NCOR2 (PNFs and NF1-MPNSTs) kDa:270**

300kD→
250kD→

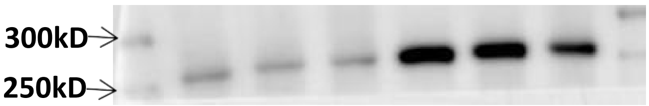

**β -actin (PNFs and NF1-MPNSTs) kDa:45**

50kDa→
40kDa→

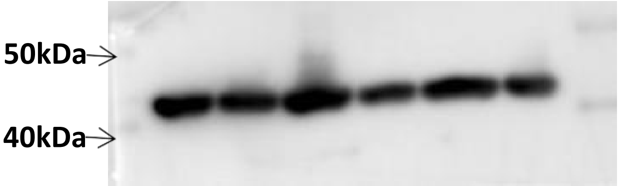

**Figure 2A**

**NCOR2 (One HSC, three PNF cell lines, four NF1-MPNST cell lines and one sporadic MPNST cell line)kDa:270**

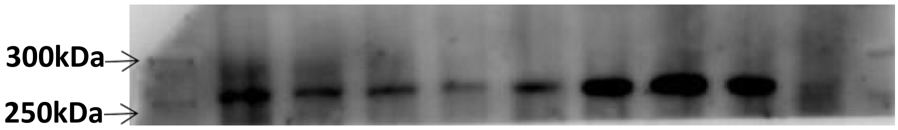


**β -actin (One HSC, three PNF cell lines, four NF1-MPNST cell lines and one sporadic MPNST cell line)kDa:45**

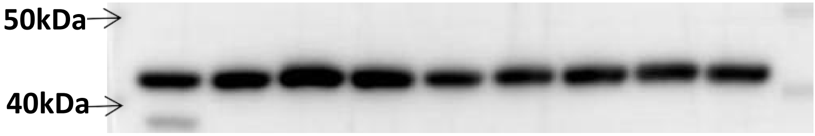


**Figure 2B**

**NCOR2 (ST8814: shNC, shNCOR2-A/B)kDa:270**

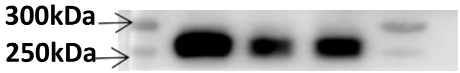


**GAPDH (ST8814: shNC, shNCOR2-A/B)kDa:37**

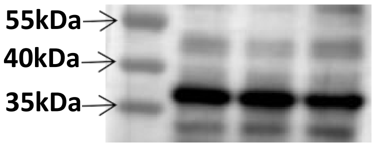


**NCOR2 (T265: shNC, shNCOR2-A/B)kDa:270**

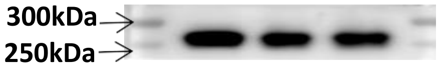


**GAPDH (T265: shNC, shNCOR2-A/B)kDa:37**

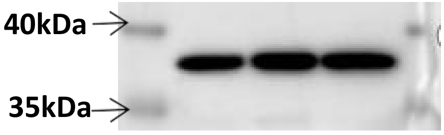


**Figure 2G**

**CDK6 (ST8814: shNC, shNCOR2; T265: shNC, shNCOR2) kDa:36**

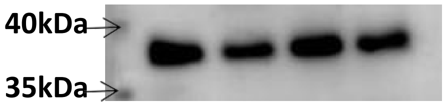


**CDK4 ( ST8814: shNC, shNCOR2) kDa:34**

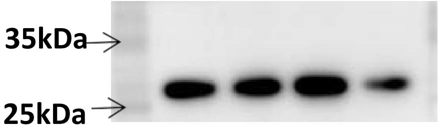


**CDK4 (T265: shNC, shNCOR2) kDa:34**

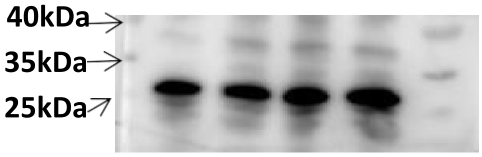


CyclinD1 (ST8814: shNC, shNCOR2; T265: shNC, shNCOR2) kDa:36

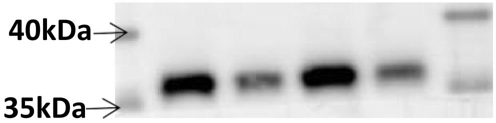


GAPDH (ST8814: shNC, shNCOR2; T265: shNC, shNCOR2) kDa:37

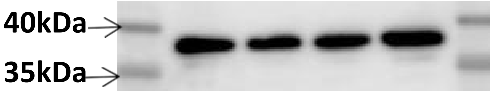

**Figure 3A**

**NCOR2 (ST462TY: shNC, shNCOR2-A/B) kDa:270**

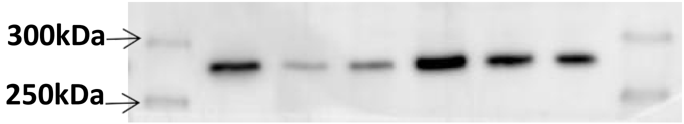


**GAPDH (ST462TY: shNC, shNCOR2-A/B) kDa:37**

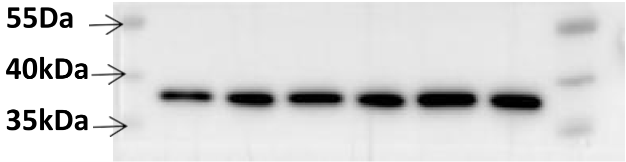

**Figure 4E**

**pERK (ST8814: shNC, shNCOR2; T265: shNC, shNCOR2) kDa:42,44**

40kDa→
35kDa→

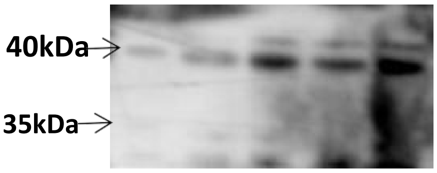

**ERK (ST8814: shNC, shNCOR2; T265: shNC, shNCOR2) kDa:42,44**

55kDa→
40kDa→

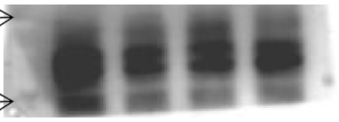

**pMEK (ST8814: shNC, shNCOR2; T265: shNC, shNCOR2) kDa:45**

55kDa→
40kDa→

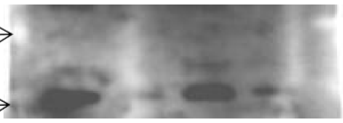

**MEK (ST8814: shNC, shNCOR2; T265: shNC, shNCOR2) kDa:45**

55kDa→
40kDa→

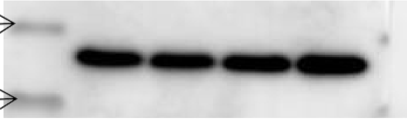

**GAPDH (ST8814: shNC, shNCOR2; T265: shNC, shNCOR2) kDa:37**

40kDa→
35kDa→

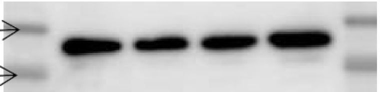

**Figure 5C**

**BDNF (ST8814: shNC, shNCOR2; T265: shNC, shNCOR2) kDa:12**

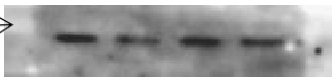


**pTrkB (ST8814: shNC, shNCOR2; T265: shNC, shNCOR2) kDa:112**

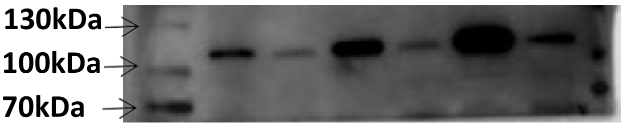


**GAPDH (ST8814: shNC, shNCOR2; T265: shNC, shNCOR2) kDa:37**

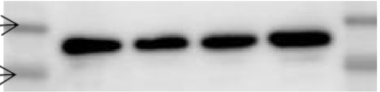


**Figure 5E**

**pMEK (T265: 0, 5, 10, -, 20, -, ng/mL BDNF) kDa:45**

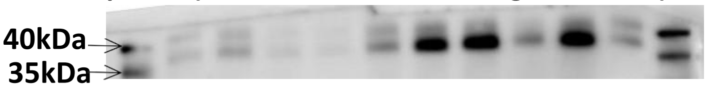


**pERK (T265: 0, 5, 10, 20 ng/mL BDNF) kDa:42,44**

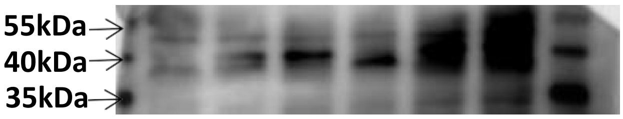


**GAPDH (T265: 0, 5, 10, 20 ng/mL BDNF) kDa:37**

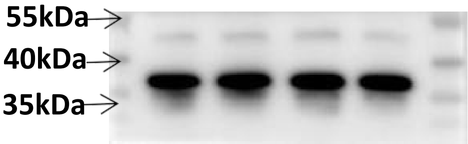

Supplement: Supplementary file 1 [file cancers-14-05798-s001.zip › Supplementary File S1.pdf]
